# Apparent Treatment-Resistant Hypertension and Cardiovascular Risk in Hemodialysis Patients: Ten-Year Outcomes of the Q-Cohort Study

**DOI:** 10.1038/s41598-018-37961-1

**Published:** 2019-01-31

**Authors:** Shigeru Tanaka, Toshiharu Ninomiya, Hiroto Hiyamuta, Masatomo Taniguchi, Masanori Tokumoto, Kosuke Masutani, Hiroaki Ooboshi, Toshiaki Nakano, Kazuhiko Tsuruya, Takanari Kitazono

**Affiliations:** 1 Department of Internal Medicine, Fukuoka Dental College, Fukuoka, Japan; 2 Department of Epidemiology and Public Health, Graduate School of Medical Sciences, Kyushu University, Fukuoka, Japan; 3 Department of Medicine and Clinical Science, Graduate School of Medical Sciences, Kyushu University, Fukuoka, Japan; 4 Fukuoka Renal Clinic, Fukuoka, Japan; 5 Department of Nephrology and Rheumatology, Faculty of Medicine, Fukuoka University, Fukuoka, Japan; 6 Department of Nephrology, Nara Medical University, Nara, Japan

## Abstract

There has been limited data discussing the relationship between apparent treatment-resistant hypertension (ATRH) and cardiovascular disease risk in patients receiving maintenance hemodialysis. We analyzed data for 2999 hypertensive patients on maintenance hemodialysis. ATRH was defined as uncontrolled blood pressure despite the use of three or more classes of antihypertensive medications, or four or more classes of antihypertensive medications regardless of blood pressure level. We examined the relationships between ATRH and cardiovascular events using a Cox proportional hazards model. The proportion of participants with ATRH was 18.0% (539/2999). During follow-up (median: 106.6 months, interquartile range: 51.3–121.8 months), 931 patients experienced cardiovascular events including coronary heart disease (n = 424), hemorrhagic stroke (n = 158), ischemic stroke (n = 344), and peripheral arterial disease (n = 242). Compared with the non-ATRH group, the ATRH group showed a significant increased risk of developing cardiovascular disease (hazard ratio [HR]: 1.27; 95% confidence interval [CI]: 1.08–1.49), coronary heart disease (HR: 1.28; 95% CI: 1.01–1.62), ischemic stroke (HR: 1.31; 95% CI: 1.01–1.69), and peripheral arterial disease (HR: 1.42; 95% CI: 1.06–1.91) even after adjusting for potential confounders. This study demonstrated that ATRH was significantly associated with increased cardiovascular risk in hemodialysis patients.

## Introduction

Hypertension is one of the most important risk factors for cardiovascular disease, and it remains an important contributor to the global health burden^1,2^. Lowering blood pressure is associated with reduced risk of cardiovascular disease in patients with chronic kidney disease, including the dialysis population^3,4^. A meta-analysis reported that hypertensive dialysis patients are more likely to derive benefit from blood pressure lowering therapy^5^. Despite the use of multiple classes of antihypertensive drugs, dialysis patients often suffer refractory hypertension likely because of population-specific treatment resistance factors including expanded extracellular fluid volume, activation of the renin-angiotensin and sympathetic nervous systems, and imbalances between vasodilators and vasoconstrictors such as nitric oxide and endothelin^6–9^.

A recent scientific statement from the American Heart Association defined apparent treatment-resistant hypertension (ATRH) as uncontrolled blood pressure with concurrent use of three antihypertensive medication classes, or four or more antihypertensive medication classes regardless of blood pressure level^10^. Accumulating epidemiological evidence shows that the prevalence of ATRH in patients with hypertension is approximately 3–30%^11^, and that ATRH is a powerful risk factor for cardiovascular disease in the general population^12–14^. The relationship between ATRH and cardiovascular disease has also been investigated in patients with chronic kidney disease. However, most studies were conducted in predialysis patients^15,16^. Therefore, there is not enough evidence to clarify the influence of ATRH on cardiovascular disease incidence in hemodialysis patients at this time. Considering the high prevalence of refractory hypertension in hemodialysis patients, it is important to clarify the precise relationship between ATRH and cardiovascular disease risk in this population. In this study, we analyzed data from the Q-cohort study, which is a longitudinal observational cohort of maintenance hemodialysis patients. The purpose of our study was to examine the association between ATRH and cardiovascular disease incidence among hemodialysis patients.

## Methods

### Study population

**Figure 1:**
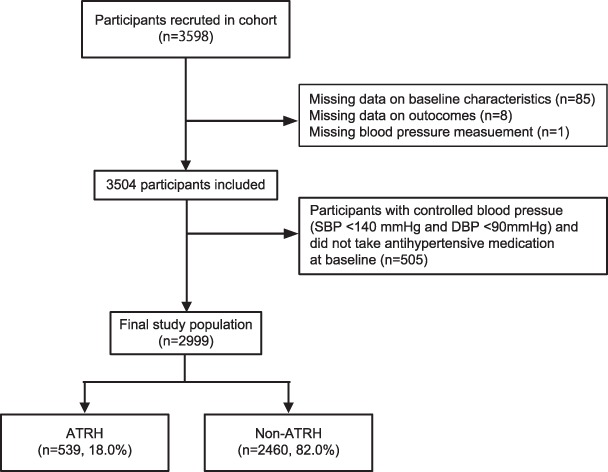
Flow diagram of the exclusion criteria to determine the study population.

The details of the design of the Q-cohort study are described elsewhere^17^. For our study, we recruited 3598 outpatients aged 18 years or older undergoing hemodialysis in 39 dialysis facilities between 31 December 2006 and 31 December 2007. Participants were followed prospectively until 31 December 2016. Exclusion criteria to determine the final analysis population are shown in the flow diagram in Fig. 1. We excluded participants with missing data on one or more baseline characteristics (n = 85) and blood pressure measurements (n = 1), and eight patients whose outcome information could not be obtained. Finally, after excluding 505 patients without a diagnosis of hypertension (systolic blood pressure [SBP] <140 mmHg and diastolic blood pressure [DBP] <90 mmHg), we enrolled the remaining 2999 patients in the final study population. The study protocol was approved by the Clinical Research Ethics Committee of the Institutional Review Board at Kyushu University (Approval Number 20–31) and all participating institutions. Written informed consent was obtained from all participants at the start of the study. The present study was performed according to the Ethics of Clinical Research (Declaration of Helsinki). The ethics committee of all participating institutions granted approval to waive requirement for written informed consent in additional follow-up surveys from 2011–2016 because of the retrospective nature of this study, which is registered in the University Hospital Medical Information Network (UMIN) clinical trial registry (UMIN ID: 000000556).

### Covariates

The main exposure was the presence of ATRH at baseline, and potential confounders were age; sex; dialysis vintage; presence of diabetes mellitus; history of cardiovascular disease; predialysis systolic blood pressure; predialysis diastolic blood pressure; serum albumin, corrected calcium, phosphorus, cholesterol, and C-reactive protein levels; Kt/V, normalized protein catabolic rate (nPCR); body mass index (BMI); use of antihypertensive agents; and dosage of erythropoiesis-stimulating agents (ESA) (per 1000 U/week increment). The ESA dosage for darbepoetin alfa administration was calculated by multiplying the dosage (µg) of darbepoetin alfa by 200. These data were collected by reviewing medical records. Blood samples were collected before starting dialysis. Controlled blood pressure was defined as SBP <140 mmHg and DBP <90 mmHg. ATRH was defined as uncontrolled blood pressure with concurrent use of three antihypertensive medication classes, or four or more antihypertensive medication classes regardless of blood pressure level^10^. Serum concentrations of albumin, calcium, phosphorus, and C-reactive protein were determined using standard methods^17^. The corrected serum calcium concentration was calculated depending on the serum albumin concentration based on Payne’s formula: corrected calcium (mg/dL) = observed total calcium (mg/dL) + (4.0 − serum albumin concentration [g/dL]).

### Outcomes

The primary outcome was cardiovascular disease (CVD) incidence, which was defined as the development of coronary heart disease (CHD), stroke, myocardial infarction, subarachnoid hemorrhage, and/or peripheral arterial disease (PAD). CHD was defined as myocardial infarction, hospitalization for unstable angina, and/or coronary intervention (coronary artery bypass surgery or angioplasty). Stroke was defined as a persistent symptomatic neurological deficit diagnosed using brain imaging. PAD was defined as critical limb ischemia requiring amputation or revascularization. The secondary endpoint was a composite of cardiovascular death, non-fatal CHD and non-fatal stroke (3-point major cardiovascular events, MACE). Participants’ health status was checked annually by local physicians at each dialysis facility. When the patient moved to another dialysis facility where a collaborator of this study was not present, we conducted follow-up surveys by mail or telephone. The events were determined on the basis of patients’ medical records.

### Statistical analysis

Group differences in continuous variables were determined using the t-test; categorical variables were compared using the chi-square test. The incidence rates and 95% confidence intervals (95% CI) for mortality and cardiovascular events were calculated using the person-year method. Poisson models were fitted to estimate incident rate ratios (IRR) and 95% CI for each event. Multivariate-adjusted hazard ratios (HR) with 95% CI for each risk factor for developing cardiovascular events were calculated using a Cox proportional hazards model. The proportional hazards assumption was assessed graphically by examining plots of log(−log survival time) and tested using analysis of Schoenfeld residuals^18^. Furthermore, we also tested the functional form of continuous covariates in the fitted Cox models using Martingale residual analysis^19^. All models were adjusted for potential risk factors for cardiovascular outcomes including age; sex; dialysis vintage; presence of diabetes mellitus; history of cardiovascular disease; serum levels of albumin, corrected Ca, phosphorus, total cholesterol, and log-transformed C-reactive protein; Kt/V; nPCR; BMI; and ESA dosage. Statistical analyses were conducted using the SAS software package (ver. 9.3; SAS Institute, Cary, NC) and the STATA software package (ver. 14.0; Stata Corp., College Station, TX). A two-tailed P-value < 0.05 was considered statistically significant.

## Results

From a total of 3598 enrolled dialysis patients, 2999 participants (83.4%) were included in these analyses, and participants’ baseline characteristics are shown in Table 1. The prevalence of ATRH at baseline was 18.0% (n = 539), of which 34 patients (1.1%) showed controlled blood pressure on ≥4 medications, and 505 patients (16.8%) had uncontrolled blood pressure on ≥3 medications. Patients with ATRH were younger, more likely to be male, and had a greater frequency of diabetes and a history of cardiovascular disease. The ATRH group had higher mean systolic blood pressure and lower cholesterol values compared with the non-ATRH group. The ESA dosage was also significantly higher in the ATRH group. We examined the risk factors associated with ATRH at baseline, and multivariable logistic regression analysis revealed that younger age, shorter dialysis vintage, higher SBP, history of cardiovascular disease, higher ESA dosage, and lower BMI were independently associated with treatment-resistant hypertension (Table 2).

**Table 1:** Baseline Characteristics of Participants with and without ATRH.

	No ATRH (n = 2460)	ATRH (n = 539)	*P*-value
Age (years)	64.7 (56.2–73.1)	63.0 (55.9–71.9)	0.07
Gender (male) (%)	60.4	67.4	<0.01
Dialysis vintage (years)	5.0 (2.0–10.8)	4.8 (2.0–9.3)	0.07
Diabetes mellitus (%)	30.3	39.3	<0.01
History of cardiovascular disease (%)	22.5	26.5	0.05
Systolic blood pressure (mmHg)	156 (144–169)	164 (151–178)	<0.01
Diastolic blood pressure (mmHg)	77 (70–85)	80 (70–86)	0.06
Serum albumin (g/dL)	3.8 (3.6–4.1)	3.9 (3.6–4.1)	0.25
Serum corrected calcium (mg/dL)	9.4 (8.9–9.9)	9.3 (8.9–9.8)	0.33
Serum phosphorus (mg/dL)	4.9 (4.2–5.6)	4.9 (4.2–5.7)	0.28
Serum total cholesterol (mg/dL)	153 (131–179)	148 (128–172)	<0.01
Serum C-reactive protein (mg/dL)	0.13 (0.05–0.30)	0.13 (0.06–0.25)	0.21
Kt/V (single pool)	1.56 (1.42–1.71)	1.56 (1.37–1.66)	<0.01
nPCR (g/kg/day)	0.95 (0.86–1.04)	0.95 (0.84–1.04)	0.31
BMI (kg/m^2^)	21.0 (19.2–22.7)	20.8 (19.0–22.4)	0.20
ESA dosage (U/week)	3000 (1500–4500)	4500 (3000–6000)	<0.01

ATRH, apparent treatment-resistant hypertension; nPCR, normalized protein catabolic rate; BMI, body mass index; ESA, erythropoiesis-stimulating agents.

Values are given as the median (interquartile range) or percentage.

**Table 2:** Risk Factors Associated with the Presence of ATRH at Baseline.

Model	Odds Ratio (95% CI)	
	Unadjusted	Adjusted^a^
Age (per 5-year increment)	0.98 (0.94–1.01)	0.95 (0.91–0.99)
Men (vs. women)	1.34 (1.11–1.66)	1.21 (0.95–1.54)
Dialysis vintage (per 5-year increment)	0.89 (0.82–0.95)	0.90 (0.82–0.98)
Diabetes (vs. no)	1.48 (1.22–1.80)	1.16 (0.93–1.45)
History of cardiovascular disease (vs. no)	1.25 (1.01–1.55)	1.31 (1.04–1.65)
Systolic blood pressure (per 10-mmHg increment)	1.22 (1.16–1.27)	1.21 (1.16–1.27)
Serum albumin (per 1-g/dL increment)	1.11 (0.89–1.39)	1.20 (0.93–1.54)
Serum corrected calcium (per 1-g/dL increment)	0.93 (0.82–1.06)	1.02 (0.88–1.17)
Serum phosphorus (per 1-g/dL increment)	1.04 (0.96–1.12)	1.05 (0.96–1.14)
Serum total cholesterol (per 10-mg/dL increment)	0.96 (0.94–0.99)	0.98 (0.95–1.01)
Serum C-reactive protein (per 1-mg/dL increment)	0.97 (0.92–1.02)	0.98 (0.92–1.03)
Kt/V (single pool) (per 1 increment)	0.57 (0.40–0.81)	0.82 (0.53–1.28)
nPCR (per 1-g/kg/day increment)	0.86 (0.52–1.41)	0.95 (0.55–1.65)
BMI (per 1-kg/m^2^ increment)	0.98 (0.95–1.01)	0.96 (0.92–0.99)
Dosage of ESA (per 1000 U/week increment)	1.11 (1.07–1.14)	1.11 (1.07–1.15)

ATRH, apparent treatment-resistant hypertension; nPCR, normalized protein catabolic rate; BMI, body mass index; ESA, erythropoiesis-stimulating agents; CI, confidence interval.

Risk estimates were computed using a multivariable logistic regression model with binary outcomes for ATRH. ^a^Adjusted for age; sex; dialysis vintage; diabetes; history of cardiovascular disease; systolic blood pressure; serum levels of albumin, corrected calcium, phosphorus, and total cholesterol; log-transformed C-reactive protein; nPCR; Kt/V; BMI; and ESA dosage.

**Figure 2:**
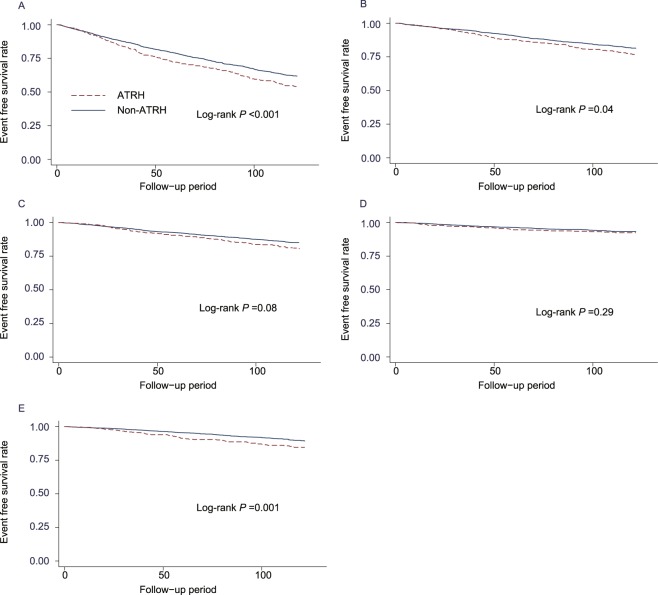
Cumulative incidence of (**A**) cardiovascular disease (CVD), (**B**) coronary heart disease (CHD), (**C**) ischemic stroke, **(D**) hemorrhagic stroke, and (**E**) peripheral arterial disease (PAD) for patients with or without apparent treatment resistant hypertension (ATRH).

The median follow-up period was 106.6 months (25–75th percentiles, 51.3–121.8 months), during which 1484 (49.5%) patients died from all-cause including 546 (18.2%) fatal cardiovascular events and 931 (31.0%) patients experienced cardiovascular events and 424 (14.1%) patients developed CHD. One thousand one hundred and eighty-three participants (39.5%) experienced MACE. The event-free survival rate of each outcome was lower among patients with ATRH compared with those without ATRH (Fig. 2). There were no significant associations between ATRH and increased risk of both of all-cause mortality (HR: 0.86; 95% CI: 0.59–1.26) and cardiovascular mortality (HR: 0.83; 95% CI: 0.63–1.10). Compared with participants without ATRH, those with ATRH had an increased risk of MACE (HR: 1.20; 95% CI: 1.04–1.39), cardiovascular disease (HR: 1.29; 95% CI: 1.11–1.51) and CHD (HR: 1.28; 95% CI: 1.01–1.61) after adjusting for age and sex (Table 3). This relationship remained essentially unchanged even after adjusting for all potential confounding factors (HR: 1.17; 95% CI: 1.01–1.35 for MACE, HR: 1.27; 95% CI: 1.08–1.49 for cardiovascular disease, and HR: 1.28; 95% CI: 1.01–1.62 for CHD). Four hundred seventy eight (15.9%) subjects developed a total stroke combining hemorrhagic and ischemic stroke. Multivariable analysis showed no significant association between ATRH and total stroke (HR: 1.21; 95% CI: 0.97–1.51). Hemorrhagic stroke occurred in 158 (5.3%) participants, and ischemic stroke occurred in 344 (11.5%). There was a significant association between ATRH and increased risk of ischemic stroke (HR: 1.31; 95% CI: 1.01–1.69) but not for hemorrhagic stroke (HR: 1.03; 95% CI: 0.70–1.52) after adjusting for potential confounding factors. PAD occurred in 242 (8.1%) participants, and ATRH was significantly associated with an increased risk of PAD (HR: 1.58; 95% CI: 1.18–2.11) after adjusting for age and sex. This relationship did not change after adjusting for all potential confounding factors (HR: 1.42; 95% CI: 1.06–1.91).

**Table 3:** Hazard Ratios for Outcomes with or without ATRH

Outcome	No ATRH (n = 2460)	ATRH (n = 539)	Hazard Ratio (95% CI)			
	Events		Unadjusted	Model 1^a^	Model 2^b^	Model 3^c^
All-cause mortality	1226 (49.8)	258 (47.8)	0.92 (0.81–1.06)	0.97 (0.85–1.11)	0.92 (0.97–1.06)	0.86 (0.59–1.26)
Cardiovascular mortality	446 (18.1)	100 (18.6)	0.98 (0.79–1.22)	1.02 (0.82–1.26)	0.94 (0.75–1.17)	0.83 (0.63–1.10)
MACE	945 (38.4)	238 (44.2)	1.18 (1.02–1.36)	1.20 (1.04–1.39)	1.16 (1.01–1.34)	1.17 (1.01–1.35)
CVD	731 (29.7)	200 (37.1)	1.28 (1.09–1.50)	1.29 (1.11–1.51)	1.25 (1.07–1.47)	1.27 (1.08–1.49)
CHD	331 (13.5)	93 (17.3)	1.28 (1.01–1.61)	1.28 (1.01–1.61)	1.24 (0.98–1.56)	1.28 (1.01–1.62)
Total stroke	374 (15.2)	104 (19.3)	1.25 (1.01–1.56)	1.28 (1.03–1.59)	1.23 (0.98–1.52)	1.21 (0.97–1.51)
Hemorrhagic stroke	125 (5.1)	33 (6.2)	1.18 (0.80–1.73)	1.16 (0.79–1.70)	1.11 (0.75–1.63)	1.03 (0.70–1.52)
Ischemic stroke	267 (10.9)	77 (14.3)	1.29 (1.00–1.67)	1.35 (1.05–1.74)	1.28 (0.99–1.66)	1.31 (1.01–1.69)
PAD	180 (7.3)	62 (11.5)	1.56 (1.17–2.08)	1.58 (1.18–2.11)	1.47 (1.10–1.96)	1.42 (1.06–1.91)

ATRH, apparent treatment-resistant hypertension; CI, confidence interval; MACE, major cardiovascular events; CVD, cardiovascular disease; CHD, coronary heart disease; PAD, peripheral arterial disease. Events are given as number (percentage).

^a^Model 1 adjusted for age and sex.

^b^Model 2 adjusted for model 1+ diabetes; history of cardiovascular disease; serum levels of albumin, corrected calcium, phosphorus, and total cholesterol; log-transformed C-reactive protein; and body mass index.

^c^Model 3 adjusted for model 2+ dialysis vintage, Kt/V, normalized protein catabolic rate, and dosage of erythropoiesis-stimulating agents.

Compared with those without ATRH, the incidence rates of clinical outcomes were higher among participants who had ATRH with uncontrolled blood pressure (Table 4). After full multivariable adjustment, participants with ATRH and blood pressure not at goal showed a significantly increased risk for each cardiovascular event except hemorrhagic stroke, all-cause or cardiovascular mortality. In participants with ATRH and blood pressure at goal, a reliable analysis to investigate the impact of ATRH on incidences of unfavorable outcomes was difficult, likely because of the small number of events.

**Table 4:** Incidence Rates (per 1000 person-years) for Outcomes According to Blood Pressure Control Status.

	No ATRH (n = 2460)	ATRH	
		Controlled (n = 34)	Uncontrolled (n = 505)
All-cause mortality, Events (%)	1226 (49.8)	16 (47.1)	242 (47.9)
Incidence rate (95% CI)	70.6 (66.7–74.6)	61.7 (37.7–101.0)	65.6 (57.1–75.3)
IRR (95% CI)^a^	1.00 (reference)	0.87 (0.54–1.43)	0.93 (0.81–1.07)
Multivariable Cox model^b^	1.00 (reference)	0.79 (0.48–1.30)	0.93 (0.80–1.06)
Cardiovascular mortality, Events (%)	446 (18.1)	5 (14.7)	95 (18.8)
Incidence rate (95% CI)	25.7 (23.4–28.2)	53.0 (21.9–127.9)	70.8 (56.7–88.3)
IRR (95% CI)^a^	1.00 (reference)	0.75 (0.31–1.81)	1.00 (0.80–1.25)
Multivariable Cox model^b^	1.00 (reference)	0.65 (0.27–1.58)	0.95 (0.76–1.19)
MACE, Events (%)	945 (38.4)	12 (35.3)	226 (44.8)
Incidence rate (95% CI)	25.7 (23.4–28.2)	53.0 (21.9–127.9)	70.8 (56.7–88.3)
IRR (95% CI)^a^	1.00 (reference)	0.89 (0.50–1.57)	1.20 (1.04–1.38)
Multivariable Cox model^b^	1.00 (reference)	0.84 (0.47–1.48)	1.19 (1.03–1.38)
CVD, Events (%)	731 (29.7)	9 (26.5)	191 (37.8)
Incidence rate (95% CI)	48.2 (44.8–51.8)	60.8 (31.5–117.4)	92.3 (78.7–108.3)
IRR (95% CI)^a^	1.00 (reference)	0.86 (0.45–1.66)	1.31 (1.12–1.53)
Multivariable Cox model^b^	1.00 (reference)	0.85 (0.44–1.65)	1.30 (1.10–1.53)
CHD, Events (%)	331 (13.5)	5 (14.7)	88 (17.4)
Incidence rate (95% CI)	20.3 (18.3–22.7)	21.1 (8.7–51.1)	26.3 (20.8–33.3)
IRR (95% CI)^a^	1.00 (reference)	1.06 (0.44–2.56)	1.33 (1.05–1.68)
Multivariable Cox model^b^	1.00 (reference)	1.20 (0.49–2.92)	1.29 (1.01–1.63)
Total stroke, Events (%)	374 (15.2)	4 (11.8)	100 (19.8)
Incidence rate (95% CI)	22.8 (20.6–25.2)	50.7 (18.9–135.7)	91.0 (73.0–113.5)
IRR (95% CI)^a^	1.00 (reference)	0.53 (0.07–3.76)	1.22 (0.83–1.80)
Multivariable Cox model^b^	1.00 (reference)	0.67 (0.25–1.80)	1.25 (1.00–1.57)
Brain hemorrhage, Events (%)	125 (5.1)	1 (2.9)	32 (6.3)
Incidence rate (95% CI)	7.3 (6.1–8.7)	3.8 (0.5–27.4)	8.9 (6.0–13.1)
IRR (95% CI)^a^	1.00 (reference)	0.72 (0.27–1.92)	1.29 (1.03–1.61)
Multivariable Cox model^b^	1.00 (reference)	0.44 (0.06–3.18)	1.07 (0.72–1.59)
Brain infarction, Events (%)	267 (10.9)	4 (11.8)	73 (14.5)
Incidence rate (95% CI)	1.61 (14.2–18.1)	16.4 (6.1–43.9)	21.1 (16.3–27.3)
IRR (95% CI)^a^	1.00 (reference)	1.02 (0.38–2.73)	1.31 (1.01–1.70)
Multivariable Cox model^b^	1.00 (reference)	1.00 (0.37–2.71)	1.33 (1.02–1.73)
PAD, Events (%)	180 (7.3)	2 (5.9)	60 (11.9)
Incidence rate (95% CI)	10.6 (9.2–12.3)	12.1 (3.0–48.9)	26.0 (19.4–34.8)
IRR (95% CI)^a^	1.00 (reference)	0.76 (0.19–3.04)	1.62 (1.21–2.17)
Multivariable Cox model^b^	1.00 (reference)	0.87 (0.21–3.57)	1.45 (1.07–1.95)

ATRH, apparent treatment-resistant hypertension; CI, confidence interval; IRR, incidence rate ratio; MACE, major cardiovascular events; CVD, cardiovascular disease; CHD, coronary heart disease; PAD, peripheral arterial disease.

Events are given as number (percentage). Controlled ATRH was defined as blood pressure at goal (systolic blood pressure <140 mmHg and diastolic blood pressure <90 mmHg) while receiving four or more classes of antihypertensive medication. Uncontrolled ATRH was defined as blood pressure not at goal while receiving three or more classes of antihypertensive medication.

^a^The IRR was calculated using a Poisson regression analysis.

^b^Hazard ratios and 95% confidence intervals were estimated using Cox’s model including the covariates for baseline characteristics (age; sex; dialysis vintage; diabetes mellitus; history of cardiovascular disease; serum albumin, corrected calcium, phosphorus, and total cholesterol; log-transformed C-reactive protein; normalized protein catabolic rate; Kt/V; body mass index; and dosage of erythropoiesis-stimulating agents).

## Discussion

The present study revealed that the prevalence of ATRH was 18.0%, and that patients with ATRH had significantly higher frequencies of male gender and diabetes, and higher SBP, lower serum cholesterol, and higher ESA dosage compared with those without ATRH, using data from a longitudinal observational cohort of maintenance hemodialysis patients. Younger age, shorter dialysis vintage, higher SBP, a history of cardiovascular disease, higher ESA dosage, and lower BMI were independently associated with ATRH. We found a strong association between ATRH and an increased risk of cardiovascular disease, CHD, ischemic stroke, and PAD. These associations remained independent even after adjusting for many potential confounders.

Epidemiological evidence suggests that ATRH is significantly associated with the development of cardiovascular disease^12–14,16,20,21^. Previous population-based cohort studies reported ATRH prevalence rates as 12.7%, 16.2%, and 18.2%^13,14,21^, and several studies of predialysis patients reported 22.9%, 26.3%, and 40.4% higher prevalence of ATRH than in the general population^15,16,22^. The ATRH prevalence of 18.0% of in our cohort was similar to that of the general population and markedly lower than reported rates for predialysis patients. Although the exact reason for this difference is unclear, we speculate that intermittent fluid removal during dialysis sessions may contribute to better blood pressure control in hemodialysis patients compared with predialysis patients.

Our results also identified the risk factors associated with ATRH in hemodialysis patients. An independent association between younger age, shorter dialysis period, and ATRH may be explained by the adverse effects of insufficient educational guidance on lifestyle habits including salt restriction^23–25^. Notably, the efficacy of reducing dry weight on blood pressure control has also been confirmed in the Dry-Weight Reduction in Hypertensive Hemodialysis Patients (DRIP) trial^26^. Also, a history of cardiovascular disease is presumed to be associated with increased atherosclerotic burden. ESA dosage was also found to be a powerful determinant of ATRH. ESA has a direct blood pressure-elevating effect regardless of its erythropoietic effect and blood rheology effect^27^. Plausible mechanisms include increased adrenergic sensitivity and changes in hemodynamics via elevated vascular endothelin-1 level and vasoactive hormone activation^28^. Also, lower BMI may reflect a patient’s poor nutritional status. With these considerations, it is reasonable to assume that malnutrition and ESA hyporesponsiveness might be closely related to the pathophysiology of ATRH in hemodialysis patients.

All-cause and cardiovascular mortality did not show a significant association with ATRH despite the increased incidence of cardiovascular events in patients with ATRH. We speculate that this may reflect a reduction in the risk of death after the onset of the event due to the effect of secondary prevention, including strengthening drug interventions and lifestyle modifications. Since secondary prevention after the onset of the cardiovascular events may reduce the risk of subsequent cardiovascular mortality, the relevance between ATRH at baseline and 10-year mortality may probably be weaken over time, which might bias the results toward the null hypothesis. Unfortunately we do not have any information after the event on changes in clinical parameters such as blood pressure, lipids or therapeutic interventions. We believe that the significance of ATRH for secondary prevention of CVD should be addressed in our future researches.

We found a significant association between ATRH and the incidence of cardiovascular disease, CHD, ischemic stroke, and PAD in our hemodialysis patients. We hypothesize that multifactorial mechanisms contribute to the increased cardiovascular disease risk among these patients. First, a possible longer period of exposure to elevated blood pressure in the ATRH group may have resulted in more severe arteriosclerosis than in non-ATRH patients secondary to accumulated blood pressure burden^29^. Second, medication nonadherence may also have increased cardiovascular risk in the ATRH group. The estimated prevalence of “pseudo-resistant hypertension”, which is elevated blood pressure secondary to white-coat hypertension, inaccurate blood pressure measurement, or medication nonadherence, is presumed to be as high as approximately 33% of resistant hypertension cases^11^. Finally, unmeasured residual confounders may explain part of the association between ATRH and cardiovascular disease. Excessive circulating aldosterone following activation of the renin angiotensin and sympathetic nervous systems, fluid retention, sleep apnea, and arterial stiffness and remodeling may contribute to the pathophysiology of ATRH^30,31^, and these conditions may be independently related to increased cardiovascular disease risk.

This study has several limitations. First, the single-time blood pressure measurement could have resulted in misclassification of participants into different blood pressure categories. Second, we did not exclude pseudo-resistant hypertension in our patient selection. This exclusion is required to determine the true ATRH prevalence and its influence on cardiovascular outcomes, and can be addressed by electronic pill bottle monitoring, pill counts or therapeutic drug monitoring, standardized blood pressure measurements, and 24-h ambulatory blood pressure monitoring. Third, we could not examine the influence of ATRH on the development of myocardial infarction due to the lack of information on a detailed list of CHD. Finally, we could not obtain data for potential secondary causes of treatment-resistant hypertension including renovascular hypertension, thyroid disease, primary aldosteronism, and sleep apnea syndrome. Despite these limitations, we believe that our findings provide useful insights that highlight the influence of ATRH on cardiovascular outcomes in maintenance hemodialysis patients.

In conclusion, our study revealed that ATRH was significantly associated with increased risks of cardiovascular disease, CHD, ischemic stroke, and PAD in hemodialysis patients. Further studies are needed to determine whether interventions to ATRH regulatory factors improve cardiovascular prognosis in maintenance hemodialysis patients.
